# Long non-coding RNA LINC01426 facilitates glioblastoma progression via sponging miR-345-3p and upregulation of VAMP8

**DOI:** 10.1186/s12935-020-01416-3

**Published:** 2020-07-20

**Authors:** Jingwei Cao, Zhanbin Tang, Zhiqiang Su

**Affiliations:** Department of Neurology, The First Affiliated Hospital of Harbin Medical University, No. 23 Youzheng Street, Nangang District, Harbin, 150001 Heilongjiang China

**Keywords:** GBM, LINC01426, Cell proliferation and growth, miR345-3p, VAMP8

## Abstract

**Background:**

Long non-coding RNAs (lncRNAs) has been extensively reported play important roles in regulating the development and progression of cancers, including Glioblastoma (GBM). LINC01426 is a novel lncRNA that has been identified as an oncogenic gene in GBM. Herein, we attempted to elucidate the detailed functions and underlying mechanisms of LINC01426 in GBM.

**Methods:**

LINC01426 expression in GBM cell lines and tissues were detected by quantitative real-time PCR (qRT-PCR). Cell Counting Kit-8 (CCK8) assays, colony formation assays, subcutaneous tumor formation assays were utilized to investigate the biological functions of LINC01426 in GBM. Dual-luciferase reporter assays, RNA immunoprecipitation (RIP) and bioinformatic analysis were performed to determine the underlying mechanisms.

**Results:**

LINC01426 is up-regulated in malignant GBM tissues and cell lines and it is capable to promote GBM cell proliferation and growth. Mechanistically, LINC01426 serves as a molecular sponge to sequester the miR345-3p and thus enhancing the level of VAMP8, an oncogenic coding gene, to promote GBM progression.

**Conclusions:**

Our results revealed the detailed mechanisms of LINC01426 facilitated cell proliferation and growth in GBM and report the clinical value of LINC01426 for GBM prognosis and treatment.

## Background

GBM is the most aggressive and common form of brain cancer in adults, it is characterized by poor survival and remarkably high tumors heterogeneity and lack of effective therapies [1, 2]. Despite advances in comprehensive treatment strategies during past decades, the median survival improved slightly [3, 4]. The major barriers to effective treatment of GBM are their high proliferation, progressive spread, and invasiveness, but the underlying mechanisms for controlling gliomas are still far from understood [5]. Therefore, there is an urgent need to study new molecular mechanisms and establish more effective and useful treatments.

LncRNAs have been shown play pivotal roles such in tumorigenesis and cancer progression, including prostate [6], colorectal [7], breast [8], bladder [9], liver [10] and brain cancers [11–13]. LncRNAs could be acted as promoter or suppressor in the development of cancer [14]. Recent reports suggested that lncRNAs. For example, lncRNA has been reported to modulate the cell proliferation and apoptosis and then contributes to glioma development [15]. Abnormal expression of lncRNA is also related to the clinical phenotype of GBM and the prognosis of patients, which can be further used as potential diagnostic or therapeutic target [16, 17]. Although some lncRNAs have been revealed obtains the clinical significance in GBM diagnosis and treatment, the detailed mechanisms remain largely unknown.

Currently, the findings of lncRNAs functionally acted as competing endogenous RNA (ceRNA) to sponge miRNA have been reported in many kinds of tumor [18–20]. miRNAs could be oncogenes or tumor suppressor genes and lots of miRNAs are involved in the development and progression of GBM [21, 22]. However, the potential lncRNA-miRNA-mRNA regulatory networks in the pathogenesis of GBM remain to be investigated.

In the current study, we screened the cancer genome atlas (TCGA) database and found a novel lncRNA LINC01426 is highly expressed in GBM tissues and associated with poor prognosis. Further analysis revealed that LINC01426 promotes cell proliferation and growth in GBM. Mechanically, we found that LINC01426 could be acted as a ceRNA to exert its oncogenic effects in GBM expression.

## Materials and methods

### Clinical specimens

The 16 cases of fresh paired GBM tissues were collected from surgical resections in the Department of digestive surgery, the first affiliated hospital of Harbin Medical University. All specimens were obtained under sterile conditions during surgery, snap-frozen in liquid nitrogen, and stored at − 80 °C. All human samples were obtained with informed consent, and the related assays were approved by the ethical review committee of the Harbin Medical University.

### Cell lines

Normal human astrocytes (NHA) and GBM cell lines U251, LN229, HS683, A172, and SW1783 were purchased from ATCC (cat#30-2008,Manassas, VA). Cell lines were cultured in standard culture conditions (37 °C, 5% CO_2_) in the culture medium recommended by ATCC.

### Construct stable cell lines

LINC01426 (cat# lnc1100768,pLVX-LINC01426) or vector (pLVX) were purchased from GeneCopoeia (cat# C0210C, Guangzhou, China), and shRNA targeting LINC01426 (cat# lnc1100768, sh#1, sh#2) or negative control (NC) were obtained from RiboBio (cat# Varies, RiboBio, China). Lentivirus was constructed in HEK-293T cells and collected from the supernatant in 24 and 48 h after transfection. These Lentiviruses were infected into U251, Hs683 and SW1783 cells, and selected by 2 μg/ml puromycin (cat# MABE343, Millipore, USA) after 48 h of infection.

### Quantitative real-time PCR (qRT-PCR)

Total RNAs were extracted from tissues and cells by TRIzol reagent (cat# 15596-026, Invitrogen, USA) following the manufacturer’s instructions. 1 µg of total RNA was reverse transcribed with the MMLV system (cat#M530B, Promega, USA). Quantitative real-time PCR (qRT-PCR) assays were performed using an ABI 7500 real-time RT-PCR system with SYBR^®^ Green Real-time PCR Master Mix (cat# PF3050SB, ABI, USA). GAPDH and U1 were regarded as the reference gene. The primers sequences are listed in Table 1. All of the CCK-8 assays were repeated three times with the similar results and data represented with mean ± SD.

**Table 1:** sequence of primers and sh RNAs

Name	Sequence (5′–3′)
Linc01426 primer	F: CGCACCCAGATACTTTTCGT
	R: GCCGTTGAGGTTGTCGTAAT
GAPDH primer	F: GTAACCCGTTGAACCCCATT
	R: CCATCCAATCGGTAGTAGCG
MiR345-3p primer	F: TAGTCCAGGGCTCGTGATGG
	R: GGGTCAGAGAGGCTGTCGAT
VAMP8 primer	F: AAGACGACATCGCAGAAGGT
	R: GACCCTCTTGGCACACATTT
CCND1	F: GCTGCGAAGTGGAAACCATC
	R: CCTCCTTCTGCACACATTTGAA
XIAP	F: AATAGTGCCACGCAGTCTACA
	R: CAGATGGCCTGTCTAAGGCAA
IL6	F: ACTCACCTCTTCAGAACGAATTG
	R: CCATCTTTGGAAGGTTCAGGTTG
IL8	F: TTTTGCCAAGGAGTGCTAAAGA
	R: AACCCTCTGCACCCAGTTTTC
MMP9	F: GGGACGCAGACATCGTCATC
	R: TCGTCATCGTCGAAATGGGC
MMP13	F: TCCTGATGTGGGTGAATACAATG
	R: GCCATCGTGAAGTCTGGTAAAAT
U1 primer	F: AGCTCATGTGCGTGATCCAG
	R: TTACACACACGGTCACTTGC
Sh-#1	AAAAGGTGCTGAACTTTGAAAATTTGGATCCAAATTTTCAAAGTTCAGCACC
Sh-#2	AAAAGGAGACGCTGTTTCACCATTTGGATCCAAATGGTGAAACAGCGTCTCC

### Western blot analysis

Indicated cells were seeded into a 6 cm plate at 5 × 10^5^ cells per plate for 48 h. Cell lysates were subjected to SDS-PAGE electrophoresis system, transferred onto a nitrocellulose membrane, and blocked with 5% skim milk in room temperature for an hour and following incubated with primary antibodies overnight at 4 °C. The next day, wash these membranes with PBS buffer and incubated with anti-mouse or rabbit IgG-HRP. The primary antibodies were listed: VAMP8 (cat#ab76021, ABCAM) and GAPDH (cat#ab8245, ABCAM), GAPDH served as a loading control. All of the western blot assays were repeated three times with the similar results and representative images were shown.

### Cellular colony formation assay

The indicated cells (1000 cells/3 ml) were seeded in the 6-well plates and incubated 14 days at 37 °C, 5% CO_2_ and the mediums were changed every 2 days. On the 14th day, the colonies were fixed and stained with crystal violet, and the number of clones per well was counted. All of the cellular colony formation assays were repeated three times with the similar results and representative images were shown, statistical analysis represented with mean ± SD.

### CCK-8 assay

Cell proliferation was measured via the Cell Counting Kit-8(CCK-8, cat# C2581, Sigma-Aldrich) assay. Briefly, indicated cells were transplanted in 96-well plates. CCK-8 solution was added to each well at 0, 24, 48 and 72 h, respectively, and then cells were incubated for 3 h. Absorbance was measured at 450 nm with a microplate reader (cat#291614, BioTek, Winooski, VT, USA). All of the CCK-8 assays were repeated three times with the similar results and data represented with mean ± SD.

### RNA-FISH assays

To detect the subcellular distribution of LINC01426, RNA FISH was carried out. Briefly, Cells were fixed with 4% PFA for 15 min, followed by permeabilization with 0.5% Triton X-100 for 5 min on ice. Then cells were subjected to incubation with RNA-FISH probes in hybridization buffer at 37 °C overnight. The nuclei were counterstained with DAPI. FISH probes (cat#C10920, RiboBio, China) were purchased from RiboBio. And the FISH assays were repeated three times with the similar results.

### RNA immunoprecipitation (RIP) assay

RIP-assay kit (cat#03-249, Millipore, USA) was used for RIP assay according to the manufacturer’s instructions. Briefly, indicated cell suspension was prepared in RIP buffer. Anti-Ago2 antibody (cat#2897S, CST, 5 μg) was incubated with the cell suspension at 4 °C overnight. Then, the precipitated RNA was purified and analyzed by qRT-PCR. Isotype-matched IgG (cat#400149, 5 μg) was used as a negative control. All of the RIP assays were repeated three times with the similar results and data represented with mean ± SD.

### Luciferase reporter assay

The wild-type or mutant interacting sequences of miR345-3p in the LINC01426 sequence or 3′-UTRs of VAMP8 were subcloned into the pmirGLO dual-luciferase plasmid (cat# E1330, Promega, Madison, WI, USA). They were named asLINC01426-WT/Mut, VAMP8-3′-UTR-WT/Mut. These vectors were co-transfected into HEK-293T cells with indicated transfection plasmids. After 48 h of co-transfection, relative luciferase activities were examined utilizing a dual-luciferase reporter assay system (cat# E1910, Promega). All of the Luciferase reporter assays were repeated three times with the similar results and data represented with mean ± SD.

### Subcutaneous tumor formation assays

2 × 10^6^ indicated stable cells were injected subcutaneously into at least 5 both flanks of BALB/c nude mice (cat# qls03-0102, Linchang biotech), and after 16 days, mice were sacrificed and photographed.

### Statistical analysis

Results are expressed as mean ± SD. The differences between groups were analyzed via Student’s *t* test or one-way ANOVA, and data were thought significantly different when P ≤ 0.05.

## Results

### LINC01426 is highly expressed in GBM and predicts poor prognosis

**Fig. 1:**
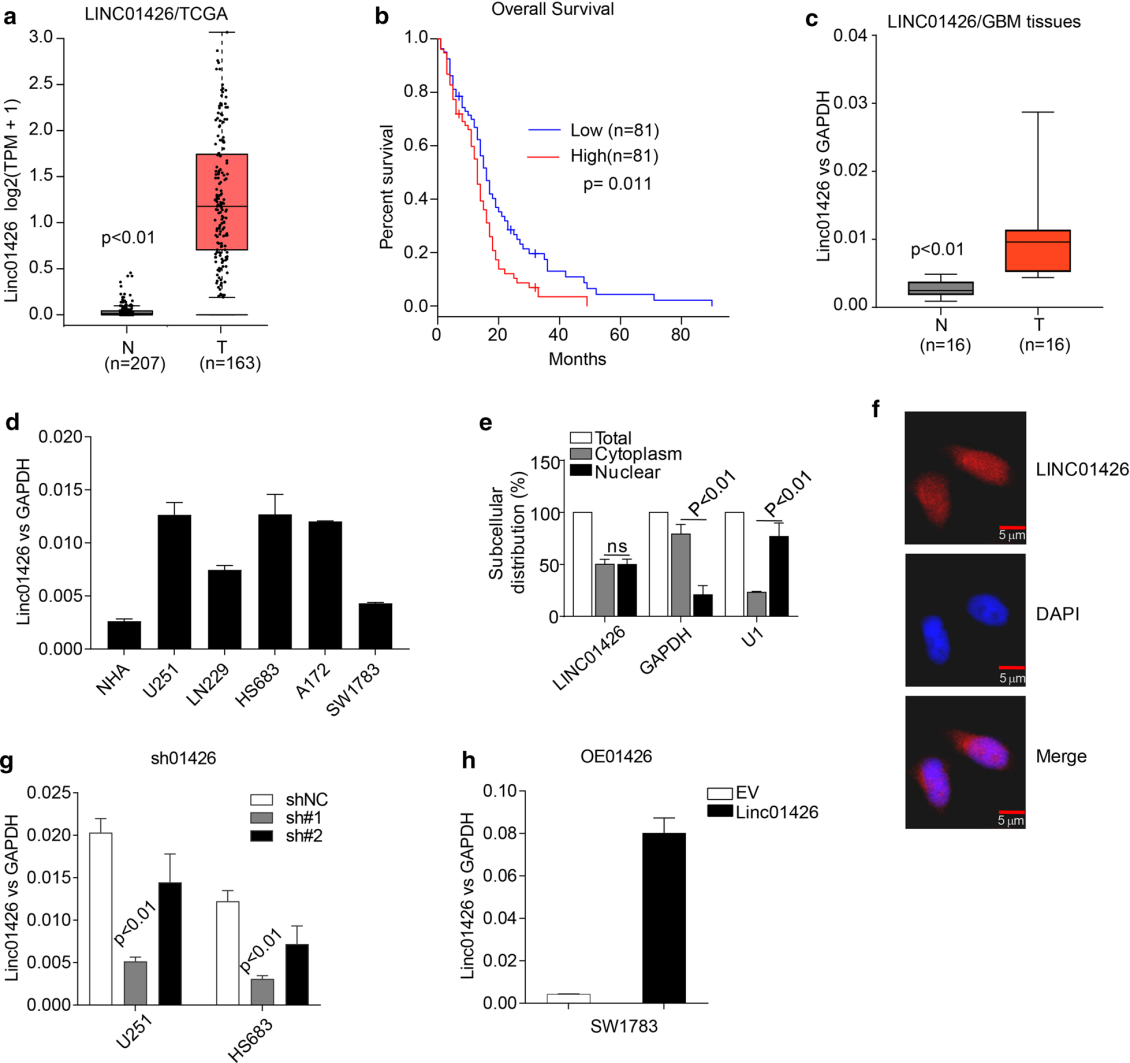
LINC01426 is highly expressed in GBM and predicts poor prognosis. **a** GEPIA analysis showed that the expression of LINC01426 is significantly elevated in GBM tissues (n = 163) compared with the normal group (n = 207). **b** The higher expression of LINC01426 predicted poor prognosis from TCGA database analysis (p = 0.011). **c**, **d** qRT-PCR was applied to access the expression of LINC01426 in 16 paired fresh GBM tissues **c** and indicated cell lines (**d**). **e**, **f** The efficiency of LINC01426 overexpression (**e**) or knockdown (**f**) in GBM cell lines. In **c**–**e** and **g**, **h**, the data are represented as mean ± SD of three times; In **f**, the experiment were repeated three times with similar results and the results of one representative experiment are shown

To identify oncogenic lncRNAs involved in GBM progression, we initially selected 20 previous reported cancer-associated lncRNAs (Additional file 1: Fig. S1) and retrieved their expression in the cancer genome atlas (TCGA)GBM patients’ cohort by an online analysis tool GEPIA (http://gepia.cancer-pku.cn/). We found that lncRNAs including LINC00511, LINC01426, GAS5, HOXA-AS2, CRNDE and DLEU1 are significantly up-regulated in GBM tissues (Additional file 1: Fig. S1). Among these highly expressed lncRNAs, only LINC01426 predicts dismal prognosis (Additional file 2: Fig. S2, Fig. 1a, b). Therefore, we examined the expression of LINC01426 in 16 fresh GBM tissues and 5 malignant cell lines, the level of LINC01426 is remarkably elevated in GBM tissues and cancer cell lines compared with normal tissues and cell lines (Fig. 1c, d).These preliminary findings suggested that LINC01426 might be an important regulator in the development of GBM and motivated us to further characterize its functions in GBM. We then detected the subcellular distribution of LINC01426 in U251 cells and found that LINC01426 is localized both in nucleus and cytoplasm (Fig. 1c, d). In order to investigate the role of LINC01426 in GBM, we silenced LINC01426 in U251 and HS683 cell lines by shRNAs (Fig. 1g) and overexpressedLINC01426 in SW1783 (Fig. 1h).

### LINC01426 regulates proliferation and growth of GBM cell lines

**Fig. 2:**
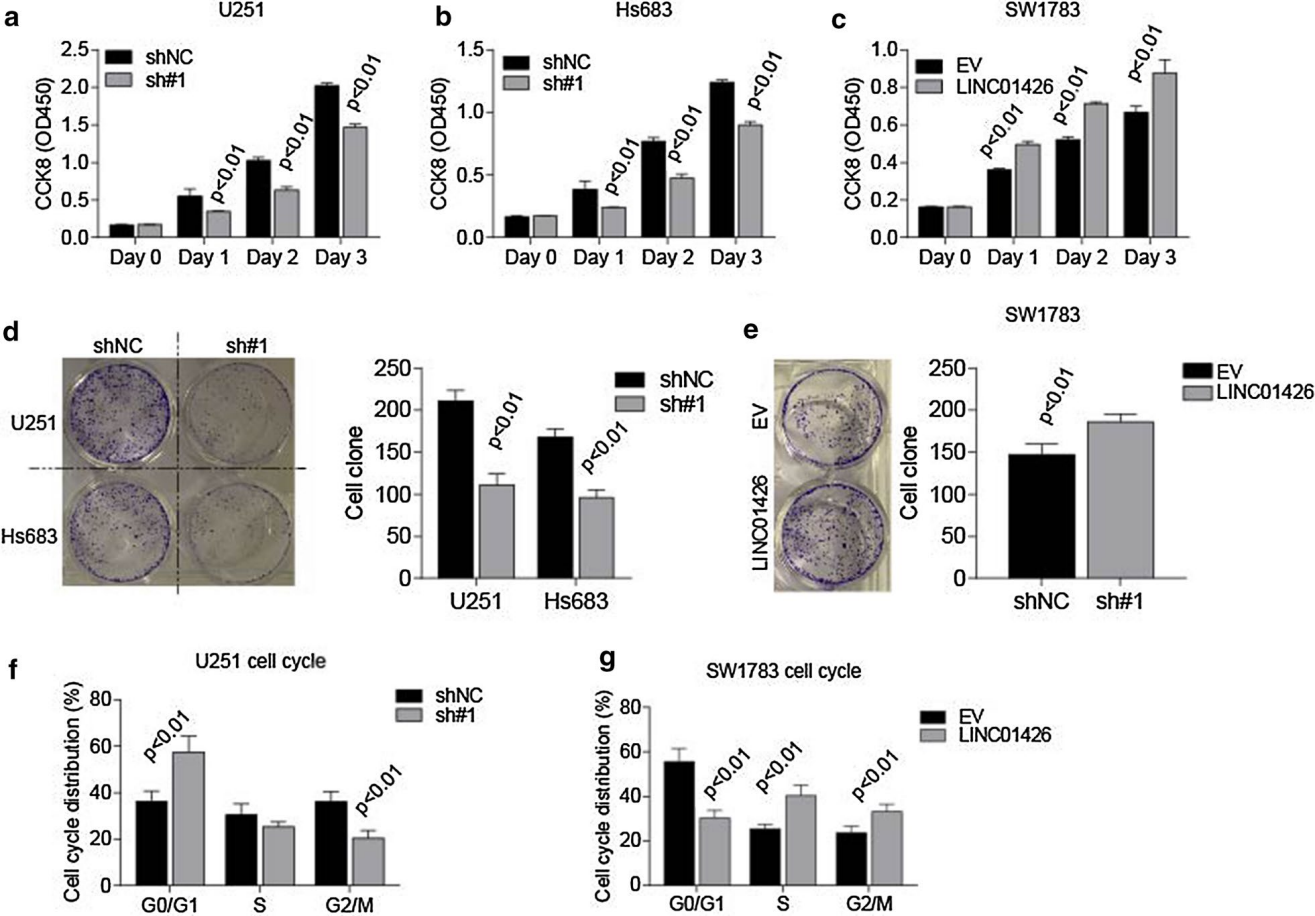
LINC01426 regulates GBM cell progression and growth. **a**, **b** CCK8 assays illustrated that silencing LINC01426 was capable to decrease the viability of U251 (**a**) and Hs683 (**b**) cell lines. **c** Overexpression of LINC01426 dramatically elevated SW1783 cell viability through CCK8 assays. **d**, **e** Colony formation assays were performed to determine the influence of Linc04126 overexpression (**d**) or knockdown (**e**) on the ability of proliferation. **f–g** FACS assays showed LINC01426 knockdown inhibits the transition of G0/G1 to S phase (**f**), while the upregulation of LINC01426 promotes the transition (**g**). all of the data are represented as mean ± SD of three times; In **d, e**, the images of one representative experiment are shown

**Fig. 3:**
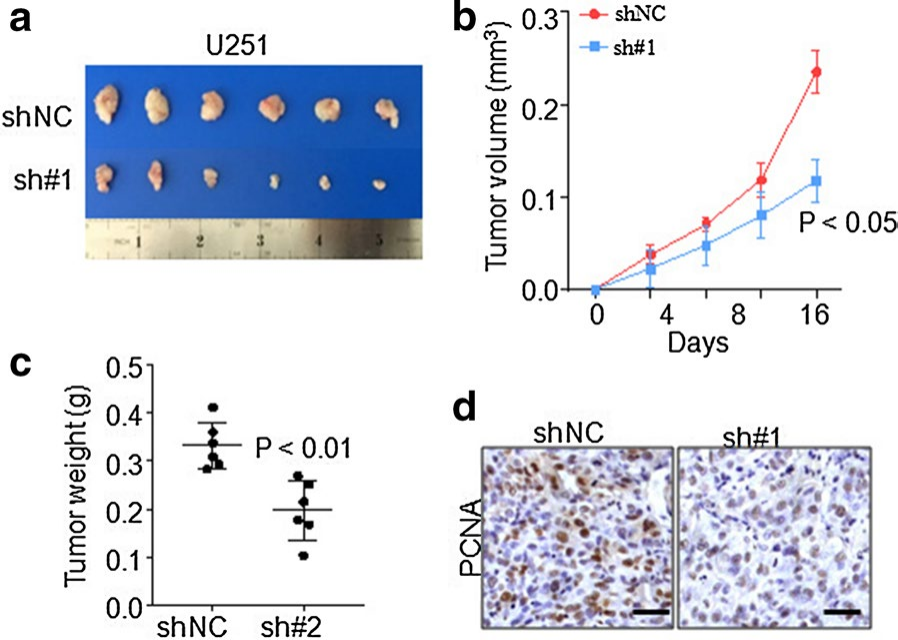
LINC01426 promotes GBM tumor formation in vivo. **a–d** Xenograft subcutaneous tumor formation assays by U251 stable cells, the tumor size (**a**), growth curve (**b**), tumor weight (**c**) and PCNA staining (**d**) are shown. Scale bar 100 μm. N = 6 in each group

According to the inhibitory efficiency, we performed our biological experiments by sh#1 in both U251 and Hs683 cell lines (Fig. 1g).The results from CCK8 cell viability and cell colony formation assays suggested that silencing of LINC01426 significantly inhibits cell proliferation and growth in U251 (Fig. 2a, d) and Hs683 cell lines (Fig. 2b, d). Accordingly, overexpression of LINC01426 promotes cell proliferation and growth in SW1783 cells (Fig. 2c, e). Besides, cell cycle analysis illustrated that knockdown of LINC01426 impaired U251 cell cycle transition from G0/G1 to S stage, while overexpression of LINC01426 promotes cell cycle from G0/G1 transit to S stage inSW1783 cell (Fig. 2f, g). In addition, subcutaneous tumor formation assays revealed that knockdown of LINC01426 impaired tumor growth in vivo (Fig. 3a, b). Both tumor weight (Fig. 3c) and PCNA staining (Fig. 3d) further confirmed the inhibitory effects by LINC01426 silencing. Collectively, highly expressed LINC01426 promotes GBM cell proliferation and tumor growth both in vitro and in vivo.

### LINC01426 is the molecular sponge of miR345-3p in GBM cell lines

**Fig. 4:**
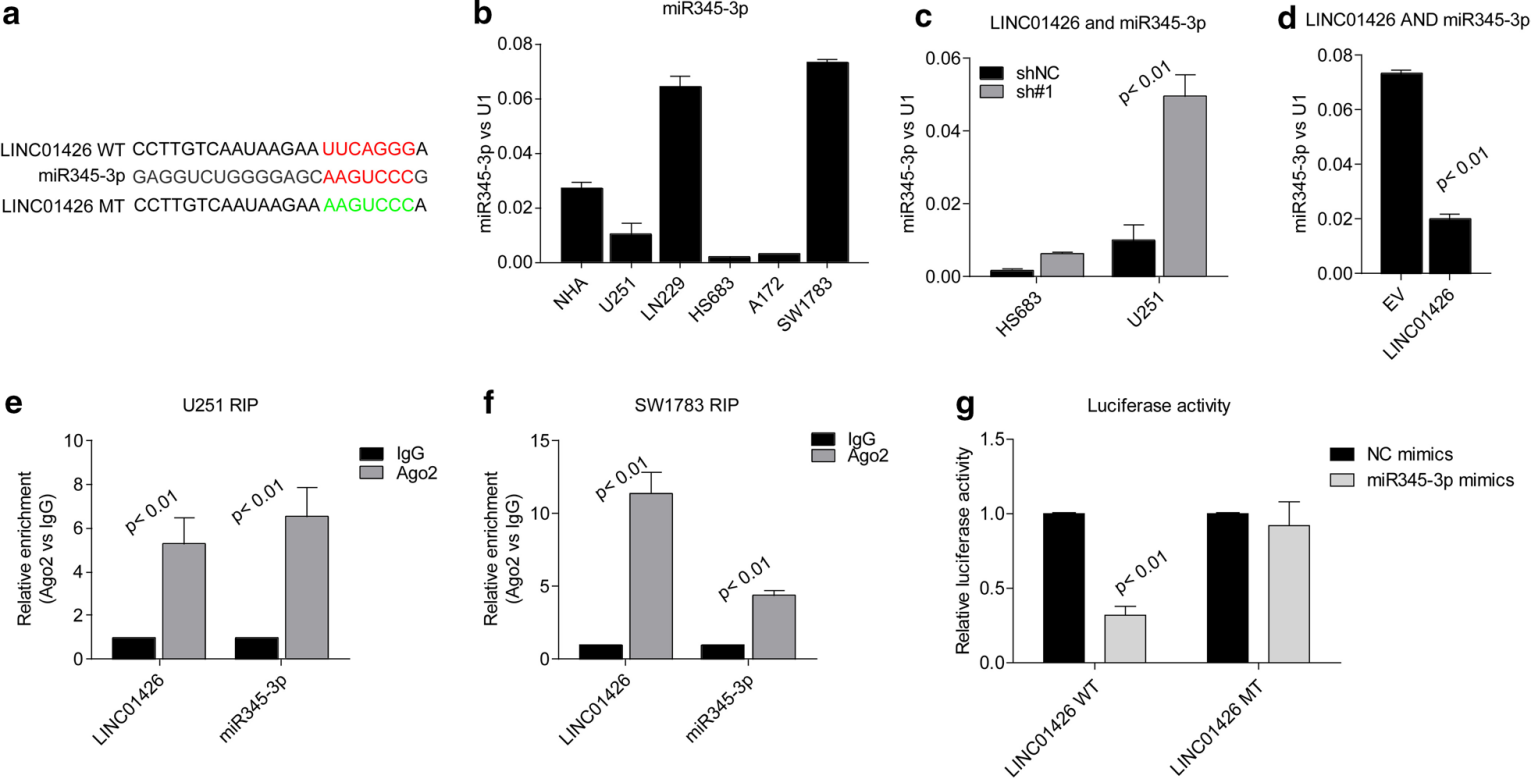
LINC01426 is the molecular sponge of miR345-3p in GBM cell lines. **a** The binding site of LINC01426 and miR345-3p was predicted by Targetscan bank. **b** qRT-PCR determine the expression of miR345-3p in GBM cell lines. **c**, **d** The miR345-3p expression level was negatively correlated withLINC01426 in HS683 and U251 (**c**) or SW1783 (**d**) stable cell lines. **e**, **f** RIP assays showed Ago2 antibody enrichment ofLINC01426 and miR345-3p in U251 (**e**) and SW1783 (**f**). **g** Luciferase reporter assays showed miR-345-3p mimics significantly repressed the LINC01426 wild type reporter viability. In **b**–**g**, the data are represented as mean ± SD of three times

In order to examine the molecular mechanism of LINC01426, we according to its subcellular distribution and hypothesized that it might act as a ceRNA in GBM cells. Both Targetscan and miRNA bank were applied to search the candidate miRNAs sequestered by LINC01426 and we found miR345-3p obtains the potential target LINC001426 (Fig. 4a). We then examined the expression of miR345-3p in GBM cell lines (Fig. 4b). The level of miR345-3p seems negatively correlated with Linc01426 in these cell lines. Furthermore, LINC01426 knockdown leads to the up-regulation of miR345-3p in HS683 and U251 cell lines (Fig. 4c) and overexpression of LINC01426 decreased its level in SW1783 cells (Fig. 4d). To further confirm the interaction between LINC01426 and miR345-3p, we performed Ago2 RIP assays in U251 and SW1783 cells. As shown in Fig. 4e, f, both LINC01426 and miR345-3p could be enriched by Ago2 suggested that they could be recruited to RNA-induced silencing complexes (RISCs) and might have functional interactions. Besides, we performed luciferase reporter assays, miR345-3p significantly repressed the wild type reporter of LINC01426, but not mutant type (Fig. 4g). In conclusion, these findings suggested that LINC01426could be act as a molecular sponge to sequester miR345-3p.

### VAMP8 is the target of miR345-3p and regulated byLINC01426

**Fig. 5:**
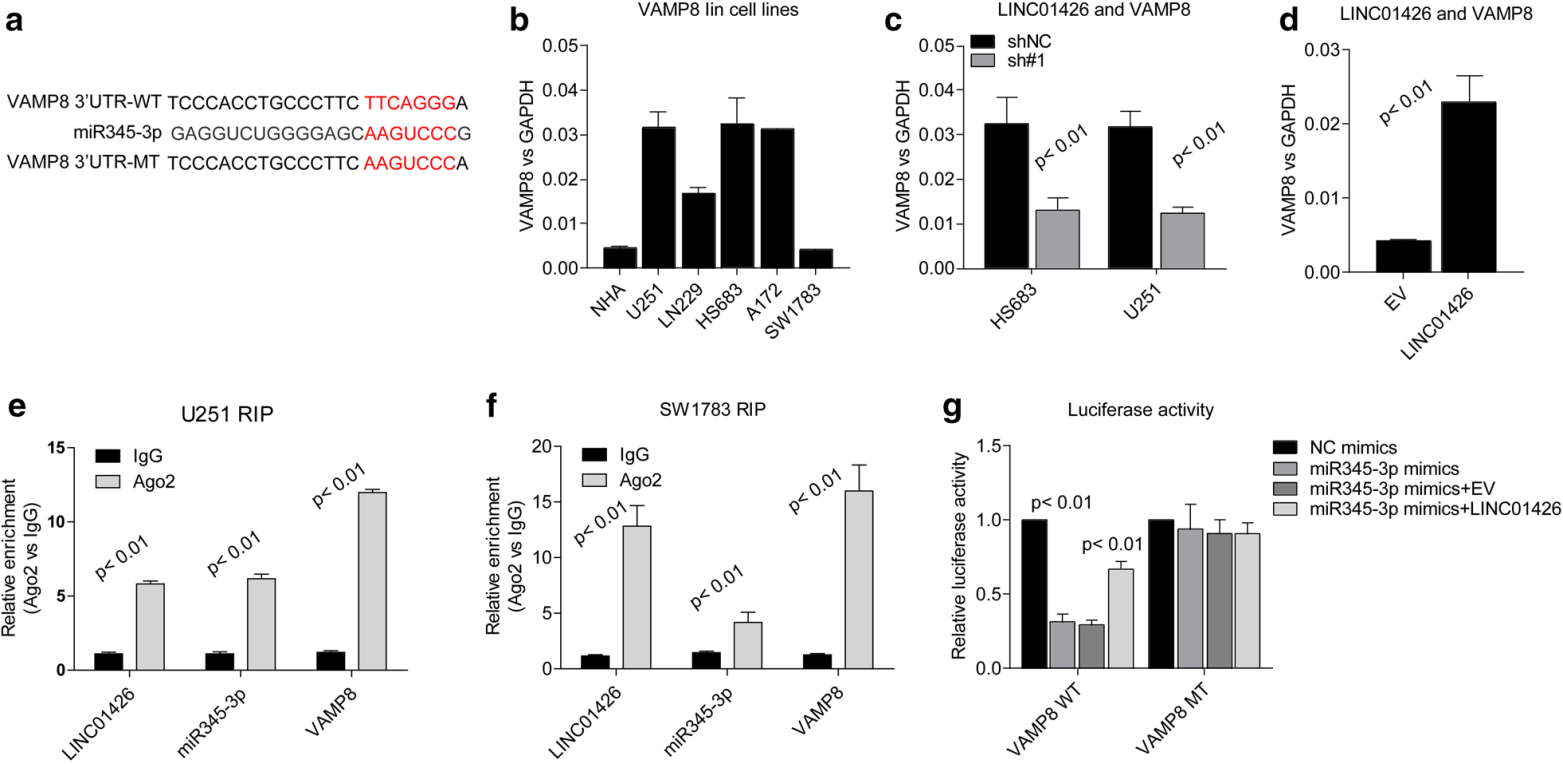
VAMP8 is the target of miR345-3p and regulated by LINC01426. **a** The binding site of VAMP8 and miR345-3p was predicted by StarBase. **b** qRT-PCR determine the expression of VAMP8 in GBM cell lines. **c**, **d** The VAMP8 expression level was positively correlated with LINC01426 in HS683 and U251 (**c**) or SW1783 (**d**) stable cell lines. **e**, **f** RIP assays using the Ago2 antibody to detect the enrichment of LINC01426, miR345-3p and VAMP8 in U251 (**e**) and SW1783 (**f**). **g** Luciferase reporter assays demonstrated the interaction between LINC01426, miR345-3p in HEK-293T cell transfected with VAMP8 wild type or mutant reporter plasmids. In B-G, the data are represented as mean ± SD of three times

Based on our preceding study on the interactionbetweenLINC01426 and miR345-3p, we proceeded to search for potential genes targeted by miR345-3p.Using online bioinformatics tools, we found thatVAMP8 is a potential target by miR345-3p (Fig. 5a).The previous study has proved that VAMP8 facilitates cellular proliferation (29).We then examined the mRNA level of VAMP8 in GBM cell lines. As shown in Fig. 5b, the expression of VAMP8 is positively correlated with LINC01426, while negatively related to miR245-3p. Moreover, knockdown of LINC01426 decreases the level of VAMP8 in HS683 and U251 cells (Fig. 5c and Additional file 3: Fig. S3), overexpression of LINC01426 significantly elevated the expression of VAMP8 (Fig. 5d, Additional file 3: Fig. S3). Furthermore, the mRNA of VAMP8 could also be enriched by Ago2 antibody in U251 and SW1783 (Fig. 5e, f). In addition, luciferase reporter assays revealed that miR345-3p significantly repressed the wild type 3′-UTR reporter of VAMP8 and the repression could be largely rescued by LINC01426 (Fig. 5g). Taken together, these results showed that LINC01426 could competitively bind to miR345-3p and then elevate the mRNA of VAMP8 in glioma cells.

### miR345-3p inhibitor and VAMP8 overexpression rescue the cell proliferation and growth inhibition caused by LINC01426 depletion

**Fig. 6:**
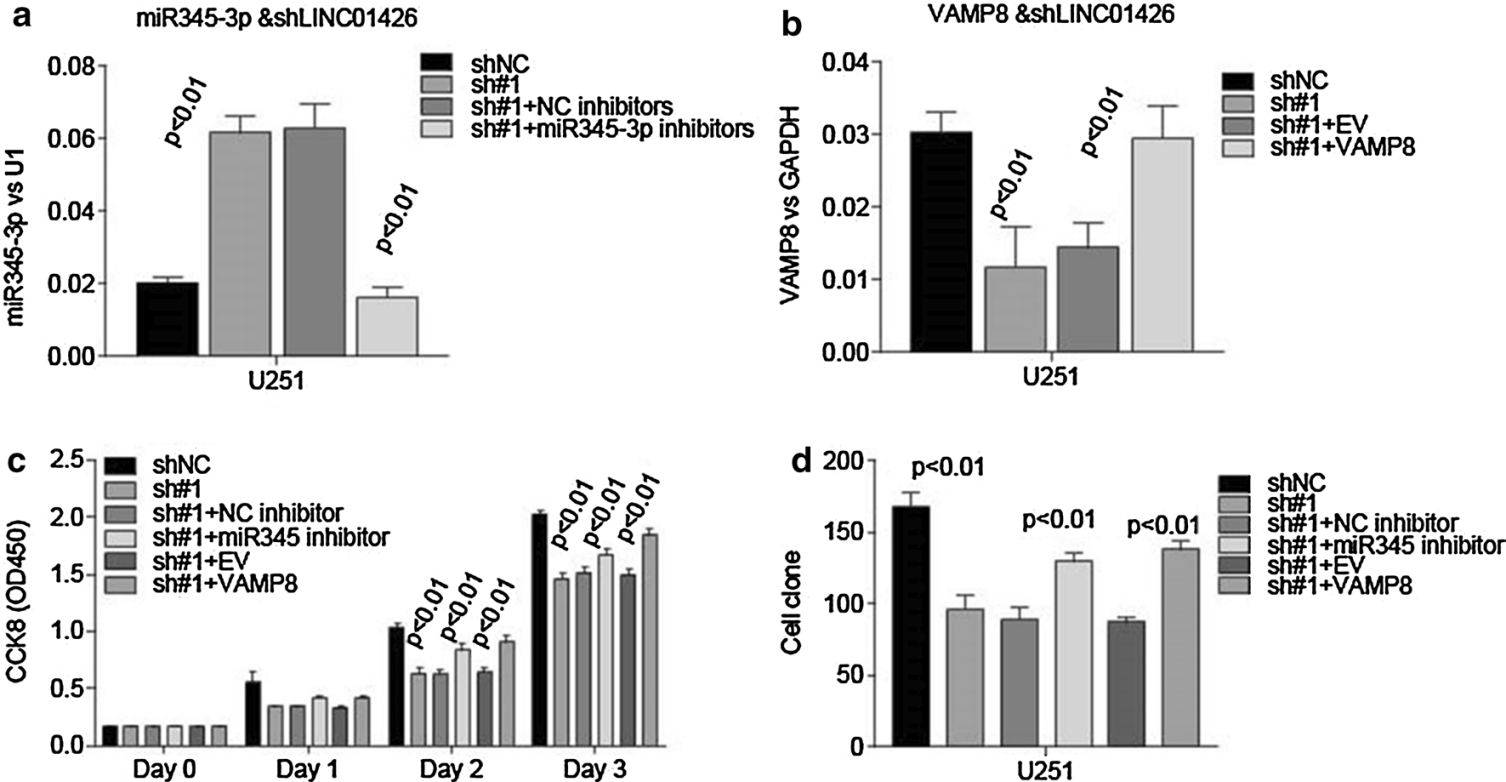
Inhibitor for miR-345-3p or overexpression of VAMP8 could rescue the cell viability and cell Colony formation caused byLINC01426 absence. **a** The miR-345-3p expression level was dramatically decreased in U251 stable cell line when treated with miR345-3p inhibitors. **b** qRT-PCR showed the overexpression efficiency of VAMP8 in the U251 cell with LINC01426 knockdown. **c** CCK8 assay showed miR345-3p inhibitors and ectopic expression of VAMP8 could rescue the impaired viability caused by LINC01426 knockdown. **c** Colony formation assay showed both miR345-3p inhibitors and ectopic expression of VAMP8 could rescue the inhibitory effect on the ability of Colony formation with LINC01426 knockdown. All of the data are represented as mean ± SD of three times

In order to elucidate that the biological effects of LINC01426 were mediated through regulating miR345-3p and VAMP8, we treated U251 cells, which were previously silenced LINC01426, with miR345-3p inhibitors (Fig. 6a). The level of miR345-3p could be inhibited by miR345-3p inhibitors. Furthermore, ectopic expression of VAMP8 in U251-sh#1 cells dramatically rescued the level of VAMP8 caused by the depletion of LINC01426. From the above, we then performed CCK8 cell viability assays and cell colony formation assays. The suppressed cell proliferation and growth could be rescued by miR345-3p inhibitors and VAMP8 overexpression in U251 cells (Fig. 6c, d). These results indicated that miR345-3p and VAMP8take part in the implementation of the regulatory functions of LINC01426 in GBM cells.

## Discussion

In this study, we found that lncRNA LINC01426 is up-regulated in GBM tissues and cell liens, the higher level of LINC01426 predicts poor prognosis. Functionally, LINC01426 was observed to promote GBM cell proliferation and growth both in vitro and in vivo. Mechanically, we revealed the dysregulated LINC01426/miR345-3p/VAMP8 axis in GBM progression. The roles and underlying mechanisms have been found here, while why LINC01426 is elevated in GBM should be further studied in the future.

As a novel biomarker, the potential role of lncRNA in predicting prognosis has been widely identified [23]. LncRNA has been found to interfere with miRNA and its downstream pathway as ceRNA, thereby affecting post-transcriptional regulation [24]. CeRNA regulatory networks are involved in many biological processes of cancer, including tumorigenesis, epithelial-mesenchymal transformation, and invasion-metastasis cascades [25]. Although the interaction between lncRNA-miRNA functional networks has attracted widespread attention in recent years [26], it remains unclear whether LINC01426 binds to miRNA to regulate GBM development. In the current study, we found that miR345-3p could be sponged by LINC01426. Previous findings revealed that miR345-3p targets TRAF6 in endothelial cells [27], while its roles in cancer development hasn’t been reported. Our results first revealed the role of miR345-3p in GBM. We further found that miR345-3p target VAMP8, a oncogenic protein in gliomas and breast cancer [28, 29], and the tumor suppressive roles could be sequestered by LINC01426.

In addition, we found that the miR345-3p inhibitor and VAMP8 overexpression could partly rescue the cell proliferation inhibition by LINC01426 knockdown in U251 cells. These findings suggested that the deregulation of LINC01426/miR345-3p/VAMP8 axis promotes GBM development. Whereas, the observations about both miR345-3p inhibitor and VAMP8 overexpression could not fully rescue the inhibitory effects by LINC0426 depletion in GBM cells reminded us that there must be some other underlying molecular mechanisms utilized by LINC01426 in GBM. Actually, we found that LINC01426 could bind to NF- κB subunits and regulate NF- κB activity in GBM cell lines (data not shown), it should be fully investigated in the future.

## Conclusions

In conclusion, we found that LINC01426 was highly expressed in GBM malignant tissues, while the high expression of LINC01426 predicts a poor prognosis. We further found that LINC01426 not only functions as a ceRNA to sponge miR345-3p and then elevated the expression of VAMP8, but also binds to NF-κB subunits and regulates its activity in GBM. These results suggest that LINC01426 plays an important role in GBM and finding out the underlying mechanism could be a potential novel strategy for the GBM treatment.

## Supplementary information

**Additional file 1: Figure S1.** Box plots depicting the expression of 20 cancer associated lncRNAs in GBM and normal tissues. Data from TCGA and analyzed on GEPIA website (http://gepia.cancer-pku.cn/index.html).

**Additional file 2: Figure S2.** Overall survival analysis of the expression of 20 cancer associated lncRNAs in GBM. Data from TCGA and analyzed on GEPIA website (http://gepia.cancer-pku.cn/index.html).

**Additional file 3: Figure S3.** Western blot analysis of VAMP8 upon knockdown or overexpression of LINC01426.

## Data Availability

All data generated or analyzed during this study are included in this published article.

## Abbreviations

GBM: Glioblastoma
LncRNA: Long non-coding RNA
VAMP8: Vesicle associated membrane protein 8
CeRNA: The competing endogenous RNA
miR345-3p: MicroRNA-345-3p
qRT-PCR: Quantitative real-time PCR
CCK-8: Cell counting kit-8
RIP: RNA immunoprecipitation
FISH: Fluorescence in situ hybridization
Ago2: Argonaute 2
