# Supplementary figures and images for: Long non-coding RNA LINC01426 facilitates glioblastoma progression via sponging miR-345-3p and upregulation of VAMP8
Source: Cancer Cell Int. 2020 Jul 20;20:327. doi: 10.1186/s12935-020-01416-3 (PMC7372762; doi:10.1186/s12935-020-01416-3)

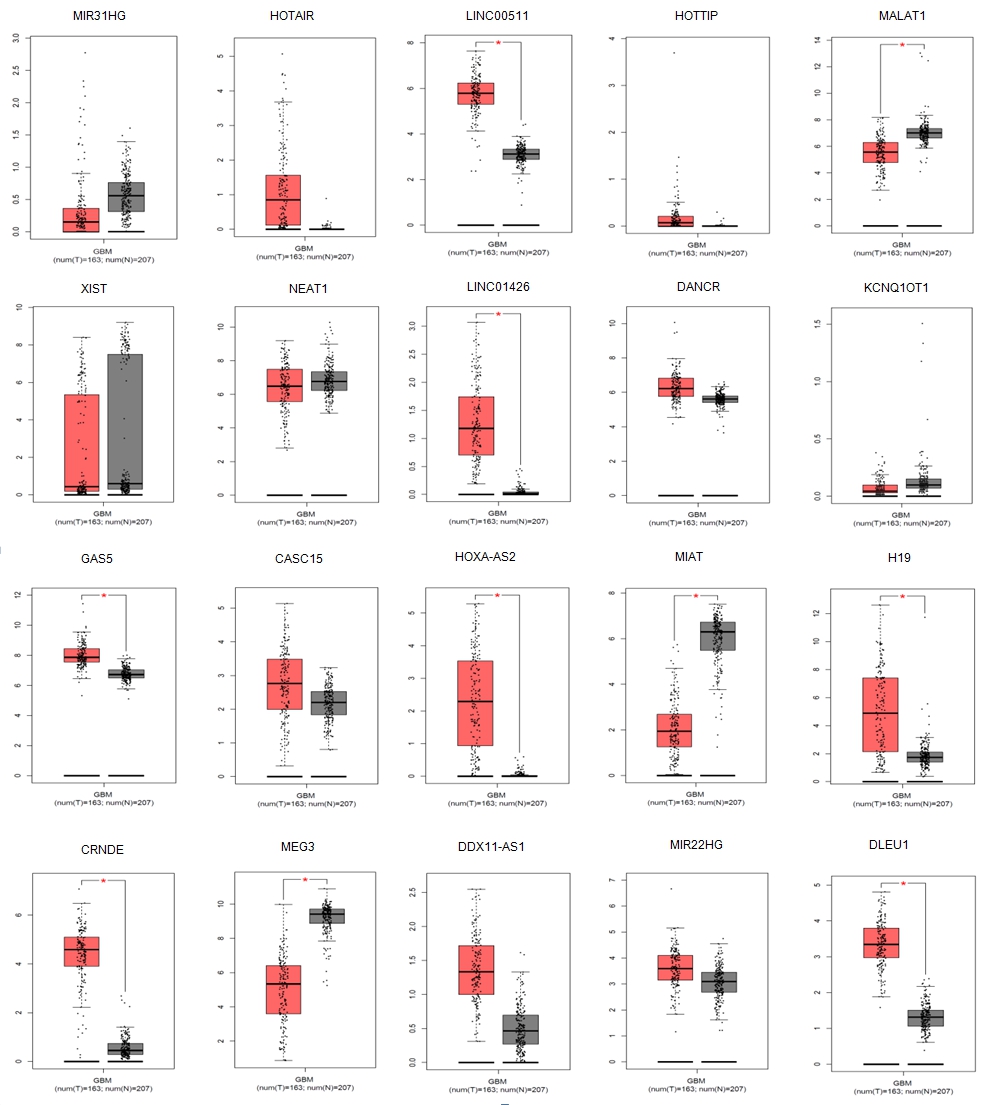

Supplement: Supplementary file 1 — Additional file 1: Figure S1. Box plots depicting the expression of 20 cancer associated lncRNAs in GBM and normal tissues. Data from TCGA and analyzed on GEPIA website (http://gepia.cancer-pku.cn/index.html). [file 12935_2020_1416_MOESM1_ESM.jpg]

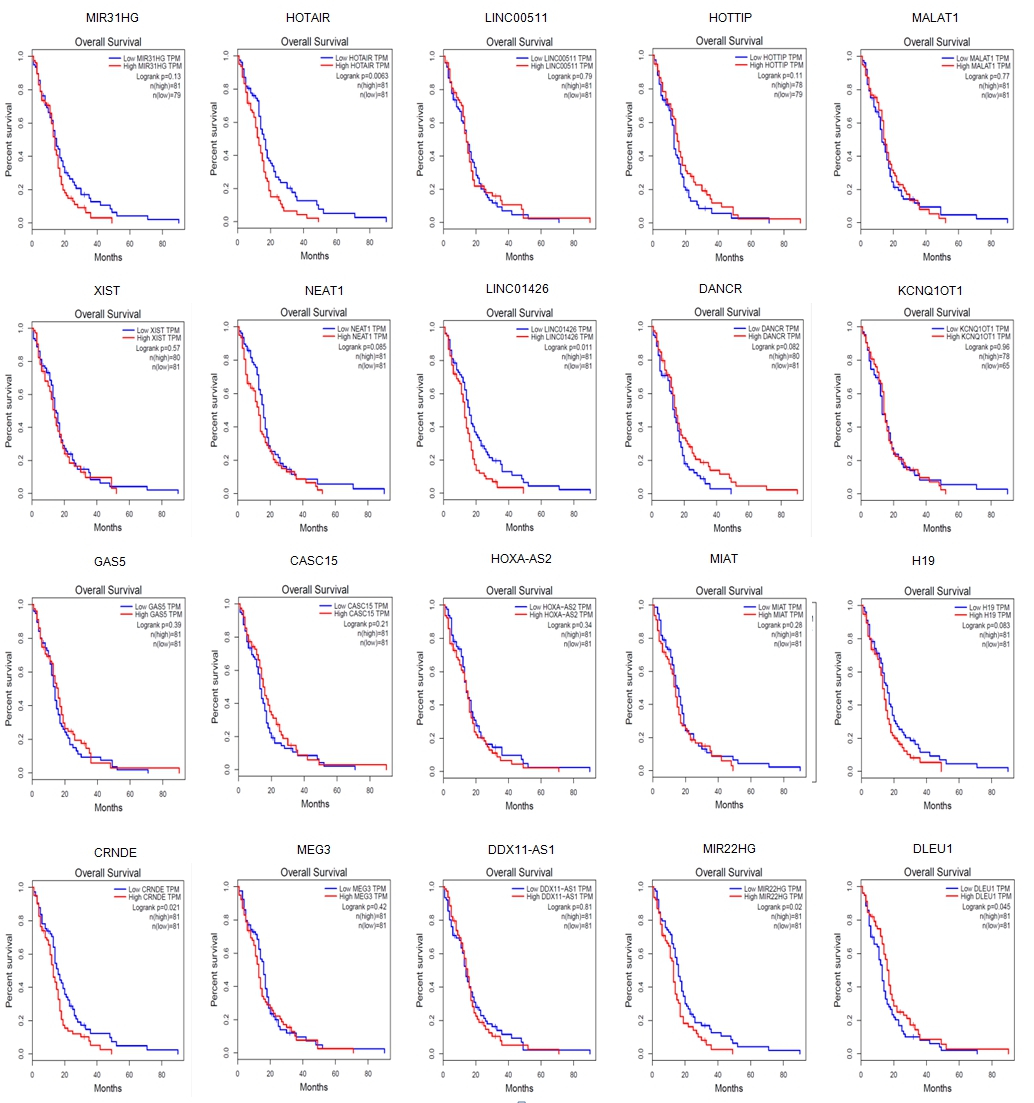

Supplement: Supplementary file 2 — Additional file 2: Figure S2. Overall survival analysis of the expression of 20 cancer associated lncRNAs in GBM. Data from TCGA and analyzed on GEPIA website (http://gepia.cancer-pku.cn/index.html). [file 12935_2020_1416_MOESM2_ESM.jpg]

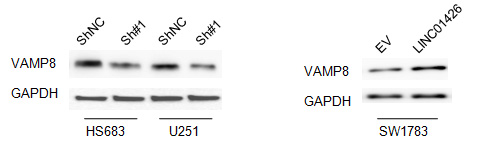

Supplement: Supplementary file 3 — Additional file 3: Figure S3. Western blot analysis of VAMP8 upon knockdown or overexpression of LINC01426. [file 12935_2020_1416_MOESM3_ESM.jpg]
